# Genome-Wide Identification, Characterization and Expression Profiling of *ADF* Family Genes in *Solanum lycopersicum* L.

**DOI:** 10.3390/genes7100079

**Published:** 2016-09-29

**Authors:** Khadiza Khatun, Arif Hasan Khan Robin, Jong-In Park, Chang Kil Kim, Ki-Byung Lim, Min-Bae Kim, Do-Jin Lee, Ill Sup Nou, Mi-Young Chung

**Affiliations:** 1 Department of Agricultural Industry Economy and Education, Sunchon National University, 413 Jungangno, Suncheon, Jeonnam 540-950, Korea; kkr@pstu.ac.bd (K.K.); salt@sunchon.ac.kr (M.-B.K.); djlee@sunchon.ac.kr (D.-J.L.); 2 Department of Horticulture, Sunchon National University, 413 Jungangno, Suncheon, Jeonnam 540-950, Korea; gpb21bau@bau.edu.bd (A.H.K.R.); jipark@sunchon.ac.kr (J.-I.P.); nis@sunchon.ac.kr (I.S.N.); 3 Department of Horticultural Science, Kyungpook National University, Daegu 702-701, Korea; ckkim@knu.ac.kr (C.K.K.); kblim@knu.ac.kr (K.-B.L.); 4 Department of Agricultural Education, Sunchon National University, 413 Jungangno, Suncheon, Jeonnam 540-950, Korea

**Keywords:** *ADF* gene, *Solanum lycopersicum*, organ-specific expression, fruit development, abiotic stress, phytohormone treatment

## Abstract

The actin depolymerizing factor (ADF) proteins have growth, development, defense-related and growth regulatory functions in plants. The present study used genome-wide analysis to investigate *ADF* family genes in tomato. Eleven tomato *ADF* genes were identified and differential expression patterns were found in different organs. *SlADF6* was preferentially expressed in roots, suggesting its function in root development. *SlADF1*, *SlADF3* and *SlADF10* were predominately expressed in the flowers compared to the other organs and specifically in the stamen compared to other flower parts, indicating their potential roles in pollen development. The comparatively higher expression of *SlADF3* and *SlADF11* at early fruit developmental stages might implicate them in determining final fruit size. *SlADF5* and *SlADF8* had relatively higher levels of expression five days after the breaker stage of fruit development, suggesting their possible role in fruit ripening. Notably, six genes were induced by cold and heat, seven by drought, five by NaCl, and four each by abscisic acid (ABA), jasmonic acid (JA) and wounding treatments. The differential expression patterns of the *SlADF* genes under different types of stresses suggested their function in stress tolerance in tomato plants. Our results will be helpful for the functional characterization of *ADF* genes during organ and fruit development of tomato under different stresses.

## 1. Introduction

The *actin depolymerizing factor* (*ADF*) gene family encodes a group of actin-remodeling proteins that are involved in the reorganization of the actin cytoskeleton. In plant cells, the actin cytoskeleton is involved in various cellular and developmental activities, such as cytoplasmic streaming, cell division, elongation, polar tip growth, and cellular organelle movement [1,2], as well as cell signaling in response to biotic and abiotic stresses [3]. A large number of actin binding proteins, including ADFs, are involved in the reorganization of the actin cytoskeleton. ADF proteins regulate the assembly of globular and filamentous actin to facilitate responses to developmental and environmental stimuli. ADF protein was first isolated from the brains of chick embryos [4] and *ADF* genes have been identified from a wide range of eukaryotes [5]. The twelve members of the *ADF* gene family identified in Arabidopsis and rice are grouped into several classes [6,7,8]. The ADFs are ubiquitous, highly conserved, low molecular weight (15–22 kDa) actin-binding proteins [9]. ADFs act as stimulus-responsive modulators in the actin cytoskeleton and are involved in in vitro monomer binding, monomer dissociation inhibition, and actin-filament binding and severing [9]. Several factors, such as pH, phosphatidylinositol 4,5-bisphosphate (PIP2), inorganic phosphate and actin filament-bound nucleotides (ATP or ADP), regulate the activities of ADF in cells [10,11,12]. In Arabidopsis, *ADF* family genes regulate the organization of filamentous actin and are reported to be involved in organ growth, tissue expansion and flowering [13]. *VvADF* was found to be involved in the initiation of new root formation from stem cuttings in grape [14]. The over-expression of *NtADF1* in tobacco suppressed the NtRac1 mutation, which causes in morphological changes of the pollen tube [15]. ADF proteins are involved in pollen tube growth by controlling cytoskeleton rearrangement [10,16]. Augustine et al. (2008) found strong evidence of the interaction of actin and ADFs in pollen tube growth of plants [11]. *ADF* family genes also have a vital role in response to various abiotic and biotic stresses. For example, accumulation of *TaADF* was found in wheat cultivars that were tolerant to freezing temperatures but was absent in sensitive cultivars [1]. Deng et al. (2010) suggested the possible involvement of *HbADF* in the latex regulation and wound plugging in *Hevea brasiliensis* [17]. *ADFs* in Arabidopsis (*AtADF2* and *AtADF4*) and barley are related to resistance against pathogens [18,19,20]. The *AtADF4* gene in Arabidopsis participates in mitogen-activated protein kinase signaling and activation of gene-for-gene resistance by regulating the cytoskeletal dynamics and transcription of the R-gene [21].

To date, no studies have investigated the organ-dependent expression profiles of *ADF* genes in vegetable crops. Tomato (*Solanum lycopersicum*) is an economically important fruit and vegetable crop world-wide. As the *ADF* gene family is known to be involved in growth, development and defense in plants, we sought to investigate the function of *ADF* genes in tomato. In the present study, we used the Sol Genomics databases to identify 11 *ADF* genes, compare them with other plant *ADF* genes, and determine their phylogenetic classification. We analyzed the differential expressions of these *ADF* genes in different organs, including different developmental stages of tomato fruit, as well as in response to different abiotic and phytohormone stresses to investigate their specific functions in tomato.

## 2. Materials and Methods

### 2.1. Identification and Analysis of ADF Family Genes

We identified 11 *ADF* genes in tomato using the InterPro domain accession number IPR002108 (representing a protein family containing the actin-depolymerising factor homology domain, the ADF-H domain) and by BLAST searches of nucleotides in the Sol Genomics Network. The genomic and corresponding protein sequences of the 11 *ADF* genes were also identified from the Sol Genomics Network (Tomato Genome protein sequences, ITAG 2.40) (http://solgenomics.net/). The primary structure of the *ADF* proteins was analyzed using the ExPASy-ProtParam tool (http://expasy.org/tools/protparam.html). The SMART web tool (http://smart.embl.de/smart/set_mode.cgi?GENOMIC=1) was used to identify and confirm the presence of the ADF-H domain. The NCBI BLAST search tool (http://www.ncbi.nlm.nih.gov/BLAST/) was used to determine the similarity between the tomato ADF proteins and those from other plants. The NCBI protein BLAST tool was used to identify the similarity among the 11 tomato ADF proteins. The Genedoc multiple sequence alignment tool (http://www.nrbsc.org/gfx/genedoc/ebinet.htm) was used to align the protein sequences. The GSDS-2.0 (Gene Structure Display Sever-2.0) web tool (http://gsds.cbi.pku.edu.cn/index.php) was used to determine the exon-intron structure by aligning the CDS and genomic sequences. The ADF protein sequences of Arabidopsis, rice and cotton were collected from the NCBI database and published articles, and their accuracy was confirmed by searching the TAIR (https://www.arabidopsis.org/), RAP-DB (http://rapdb.dna.affrc.go.jp/) and Cottongen (https://www.cottongen.org/) databases, with manual corrections applied as needed. The complete protein sequences of tomato ADF proteins were aligned with other plant proteins using Clustal Omega (http://www.ebi.ac.uk/Tools/msa/clustalo/) and the phylogenetic tree was created using MEGA 6.0 in the Neighbor-Joining (NJ) algorithm method [22]. The significance of nodes in the tree was evaluated by percentage bootstrap analysis with 1000 replicates. Conserved motifs were analyzed using the MEME (Multiple Em for Motif Elicitation, V4.9.0) web tool with the following parameters: optimum motif width ≥6 and ≤200 and maximum motif number 10. The sub-cellular localization of the identified proteins was analyzed using ProtComp 9.0 from the Softberry web tool (http://linux1.softberry.com/berry.phtml). We identified putative cis-regulatory elements of about 5 to 10 bp in around 1500-bp upstream from the start codon (ATG) using the PlantCARE web tool (http://www.dna.affrc.go.jp/PLACE/signalscan.html).

### 2.2. Analysis of Gene Duplication and Chromosomal Localization

The chromosomal locations of the 11 tomato *ADF* genes (i.e., start and end positions) were identified using the SGN database (https://solgenomics.net/) and their positions along the 12 chromosomes were mapped using the MapChart software (https://www.wageningenur.nl/en/show/Mapchart-2.30.htm). The NCBI protein BLAST tool was used to find similarity between the 11 tomato *ADF* genes and the duplication analysis was performed according to Kong et al. (2013); where both the identity and aligned region of the gene sequences covered >80%, the area was defined as a segmental duplication [23].

### 2.3. Preparation of Plant Materials and Sample Collection

Seeds of tomato cv. *Ailsa craig* were germinated and the seedlings were grown in potting soil mixture at 25 °C with a 16 h light and 8 h dark cycle in a culture room at the Horticulture Department of Sunchon National University, Korea Republic. For expression analysis of *ADF* genes, fresh roots, stems and leaves were destructively harvested from seven-week-old plants. The plants were then transferred to a greenhouse to allow their further growth and development for analysis of flowers and fruits. Individual sepals, petals, stamens and ovaries were collected from flowers at the anthesis stage. Fruit samples were collected at six different stages of growth: (i) 1 cm fruit, young fruits around 2 weeks from the date of pollination and about 0.8–1.0 cm in diameter; (ii) immature (IM) fruit, around 20 days from the date of pollination and approximately 2 cm in diameter; (iii) mature green (MG) fruit, 45 days from the date of pollination; (iv) breaker (B), beginning of ripening when the green color changes to light yellow-orange; (v) (B + 5), fruit 5 days after the breaker stage; and (vi) (B + 10), fruit 10 days after the breaker stage. All samples were immediately frozen in liquid nitrogen and stored at −80 °C until RNA isolation.

Four-week-old plants with synchronized growth were selected to study the expression of *ADF* genes in response to different stress treatments. Seven different treatments, heat, cold, drought, wounding, NaCl, abscisic acid (ABA) and jasmonic acid (JA), were imposed and gene expression was measured at various time points during the treatments (0, 1, 3, 6, 12 and 24 h). To impose the heat and cold treatments, the seedlings were incubated at 40 °C and 4 °C, respectively. For the drought treatment, the plants were gently pulled from the soil, their roots were washed carefully with fresh water, and the plants were placed on a dry paper towel for 24 h. For the wounding treatment, the leaves of the seedlings, including the midrib, were cut with a scalpel. For the NaCl treatment, the roots of the seedlings were submerged in a solution containing 200 mM NaCl for the entire 24 h. For the ABA and JA treatments, seedlings were sprayed with two different concentrations of ABA and JA (100 μM and 50 μM). Samples collected at 0 h were used as a control for all stress treatments. For each treatment three samples were collected from each of three individual plants. All samples were immediately frozen in liquid nitrogen and stored at −80 °C until RNA isolation.

### 2.4. RNA Extraction and cDNA Synthesis

Qiagen RNeasy mini kits (Qiagen, Valencia, CA, USA) were used for the extraction of RNA form the different organs and stress treated samples. The Qiagen RNase free DNaseSet (Qiagen, Hilden, Germany) was used to remove any genomic DNA contaminants from the RNA. The RNA concentration in each sample was measured by a NanoDrop^®^ 1000 Spectrophotometer (Wilmington, DE, USA). Single-stranded cDNA was synthesized from the RNA with the Superscript^®^ III First-Strand cDNA synthesis kit (Invitrogen, Carlsbad, CA, USA).

### 2.5. qPCR Expression Analysis

For the qPCR expression analysis, gene-specific primers were designed for all *ADF* genes using Primer3 software (http://frodo.wi.mit.edu/primer3/input.htm) (Table S1). *EF1a* (F: TCAGGTAAGGAACTTGAGAAGGAGCCT, R: AGTTCACTTCCCCTTCTTCTGGGCAG) [24] expression was used for normalization. The qPCR was conducted in 10-μL reaction volumes consisting of 1 μL of 50 ng cDNA, 2 μL forward and reverse primers, 5 μL 2× qPCRBIO SyGreen Mix Lo-ROX (PCRBIOSYSTEMS, CA, USA,) and 2 μL double-distilled water. The reaction conditions were: 95 °C for 300 s followed by 40 cycles at 94 °C for 10 s, 58 °C for 10 s and 72 °C for 15 s. The melting temperature was set to 95 °C for 10 s, 65 °C for 60 s and 97 °C for 1 s. The amplification and Cq value of each sample was recorded using the LightCycler96 (Roche, Mannheim, Germany). The relative gene expression was calculated using the 2^−∆∆Ct^ method [25]. To determine significant changes in gene expression levels among the different time points for each treatment, MINITAB statistical software 15 (Minitab Inc., State College, PA, USA) was used to conduct the analysis of variance of the relative expression of each gene following a generalized linear model. Turkey’s pairwise comparison test was conducted for mean separation.

## 3. Results

### 3.1. Sequence Analysis and Genomic Organization of Tomato ADF Genes and Corresponding Proteins

**Figure 1:**
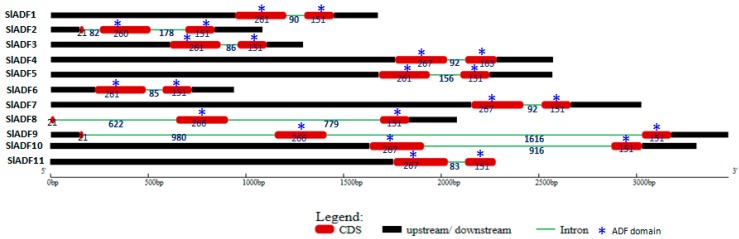
Schematic representation of the exon-intron distribution in the tomato *actin depolymerizing factor* (*ADF*) gene family. The red boxes represent the exons and the green lines represent the introns. Asterisks indicate the ADF-H domain position in the exon. The numbers represent the length of exons and introns in bp.

**Figure 2:**
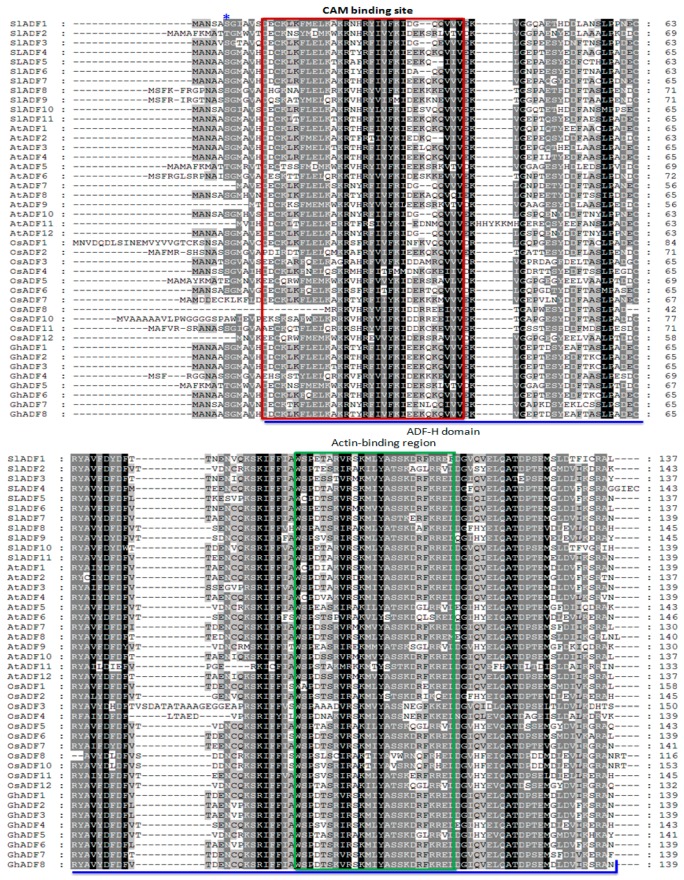
Alignment of all deduced tomato ADF polypeptides with those of Arabidopsis, rice and cotton. Asterisks indicate the putative serine phosphorylation site. The red box indicates the putative CAM binding site and the green box indicates the amino acid residues essential for actin binding. The dark black background highlights the identical amino acids and the light background highlights the amino acids with >50% identity. The blue underline represents the ADF-H domain position.

The 11 *ADF* genes that were identified in this study were designated as *S. lycopersicum ADF* (*SlADF*) and the corresponding encoded proteins were named SlADF. The predicted size of the 11 SlADFs ranged from 137–145 amino acids (aa) (Table 1). The ADF-H domain was found in all 11 tomato ADFs, beginning at 12–18 aa and ending at 137–145 aa (Table 1). The predicted isoelectric point of the SlADFs varied from 5.12 to 8.69 indicating some ADF proteins were acidic, while others were basic (Table 1). The grand average of hydropathy (GRAVY) of the SlADFs varied from −0.293 to −0.598, showing that the proteins had hydrophilic characteristics (Table 1). The subcellular localization of the proteins was predicted to be extracellular, and it is possible that all are multi-located proteins found both in the nucleus and in the cytoplasm (Table 1). Analysis of the genomic structure of the *ADFs* revealed that *SlADF2, SlADF8* and *SlADF9* contain three exons and two introns at conserved positions, with the first coding sequence was much smaller in size compared to the other two coding sequences (Figure 1). The ADF-H domains were located in the 2nd and 3rd exons of these three genes (Figure 1). The other eight *ADF* genes contained two exons and one intron and the ADF-H domain were located in both of the exons (Figure 1). The tomato ADFs shared more than 80% sequence similarity with Arabidopsis and other plant ADFs, with a range of 75% to 91% (Table S2). The sequence identity among the 11 tomato ADFs ranged from 51% to 89% (Table S3). SlADF7, SlADF5, SlADF4 and SlADF11 shared more than 80% similarity; SlADF3 shared more than 80% similarity with SlADF6; SlADF4 shared more than 80% similarity with SlADF5; and SlADF1 shared more than 80% similarity with SlADF10, indicating possible gene duplication. Alignment of the predicted tomato ADFs with the ADFs of Arabidopsis and rice revealed that the tomato proteins contained the conserved serine residue that might be the putative phosphorylation site of ADFs and a CAM (calmodulin) binding region at the N-terminus region (Figure 2). The alignment also revealed that the ADF-H domain position and the possible actin-binding region were conserved in all of the ADFs (Figure 2). Motif searches revealed that motifs 2 and 3 were characteristic of the N-terminus and motifs 1 and 4 were characteristic of the C-terminus. The putative phosphorylation site was found in motif 2, the CAM binding region was located in motif 3, and the PIP2/actin binding region was found in motif 1 (Figure S1).

### 3.2. Phylogenetic Analysis of SlADF Proteins

**Figure 3:**
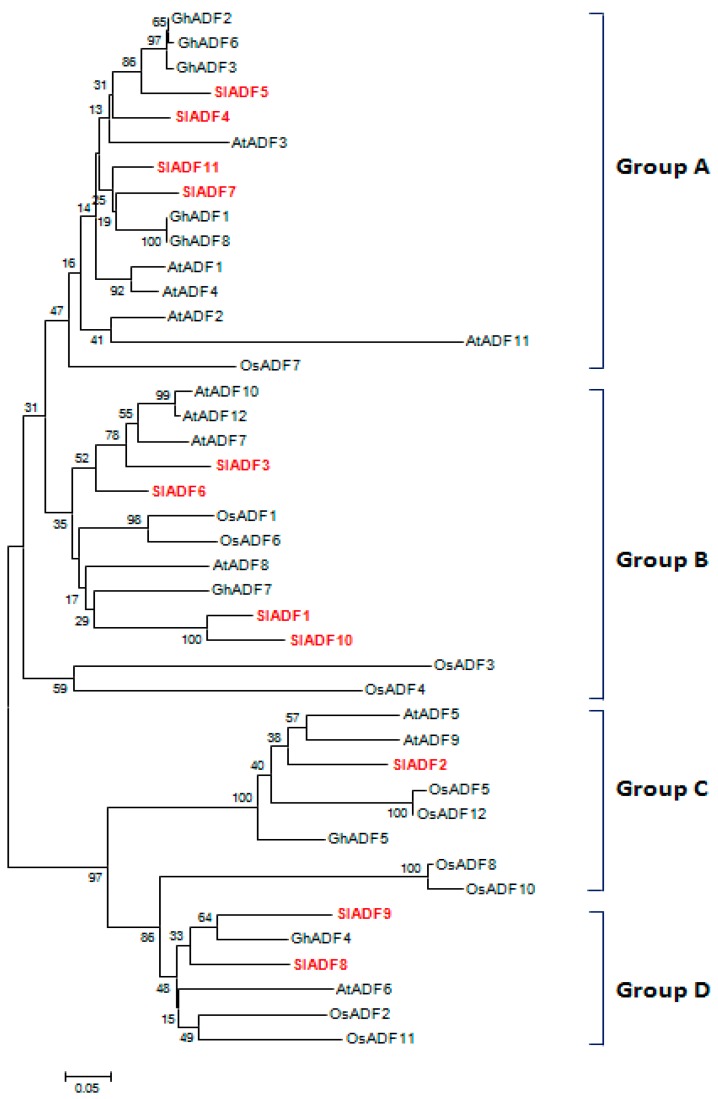
Phylogenetic tree of tomato actin depolymerizing factor (ADF) proteins with those of Arabidopsis, rice and cotton. The 11 tomato ADF proteins are shown in red. The deduced full-length polypeptide sequences were used to create the tree. The tree was constructed by the Neighbor-joining method in MEGA 6.0 software following the Poisson-model. The bootstrap values were calculated as a percentage of 1000 replicates. Bootstrap values were shown next to the branches. The scale represents the units of the number of amino acid substitutions per site. Protein sequences of Arabidopsis, rice and cotton were taken from published literature, the TAIR, RAP-DB, Cottongen and the NCBI database.

The phylogenetic tree classified the ADF proteins into four main groups (Figure 3A–D), consistent with published data and expression profiles of ancient ADF proteins [7,8]. SlADF4, SlADF5, SlADF7 and SlADF11 clustered in Group A with Arabidopsis, rice and cotton ADFs; members of group A from these other species are strongly expressed in a wide range of tissues such as root, leaf, and flower (Figure 3) [7]. SlADF1, SlADF3, SlADF6 and SlADF10 belonged to Group B along with Arabidopsis, rice and cotton ADFs that are expressed in root and reproductive tissues (Figure 3) [7]. SlADF2 clustered in Group C, and SlADF8 and SlADF9 clustered in Group D. Group C and D ADFs from other plants are expressed moderately in all tissues (Figure 3) [7].

### 3.3. Chromosomal Position and Duplication of Tomato ADF Genes

The 11 tomato *ADF* genes were manually mapped on the 12 tomato chromosomes based on MapChart results (Figure S2). The *ADF* genes were unevenly distributed, with three genes found on chromosome 9, two on chromosomes 6 and 10, and one on chromosomes 1, 3 and 4. Segmental duplication or tandem duplication during evolution are responsible for generating the different gene families. Among the 11 tomato *ADF* genes, *SlADF1*, *SlADF3*, *SlADF4*, *SlADF5*, *SlADF6*, *SlADF7* and *SlADF11* were found to be segmentally duplicated as those genes had more than 80% identity and query coverage (Figure S2 and Table S4). The duplicated genes were located on chromosomes 1, 3, 4, 5, 6, 9 and 10. None of the genes had tandem duplication.

Several cis-acting elements related to development, tissue specific expression, seed specific regulation, abiotic and biotic stress response, auxin and ethylene response, and circadian regulation were identified (HD-Zip 1, HD-Zip 2, RY-element, as-2-box, Skn-1_motif, Box-W1, TC-rich repeats, ERE, TGA-element, AuxRR-core, HSE, MBS, CE3, MRE, TCA-element, O2-site and circadian), suggesting the possible involvement of this gene family in development and stress tolerance (Table S5).

### 3.4. Expression Analysis of Tomato ADF Genes in Different Organs

**Figure 4:**
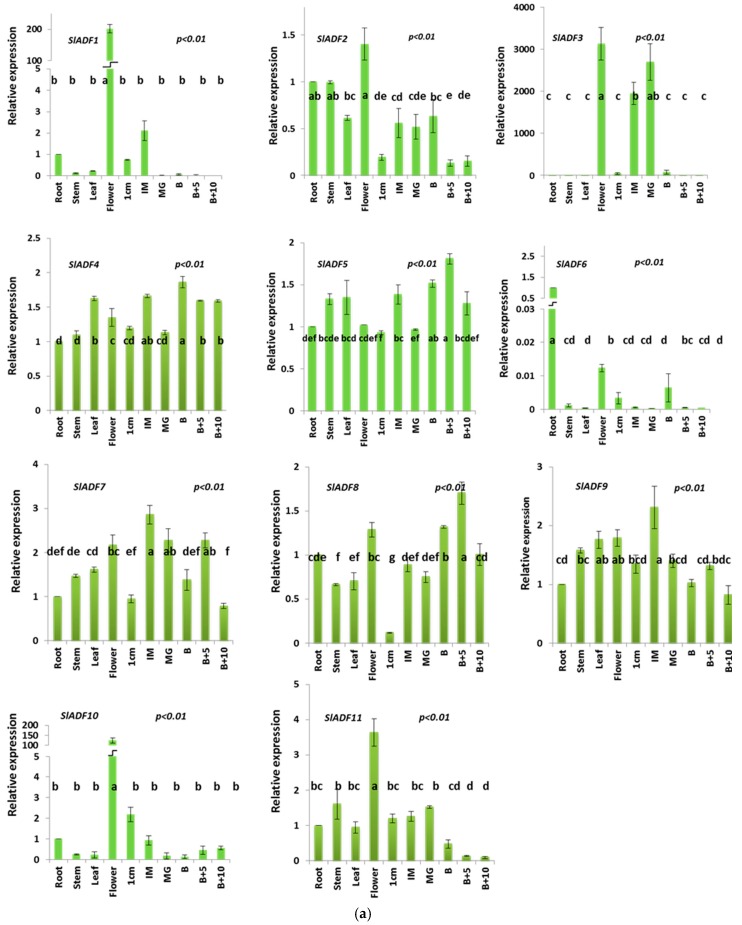

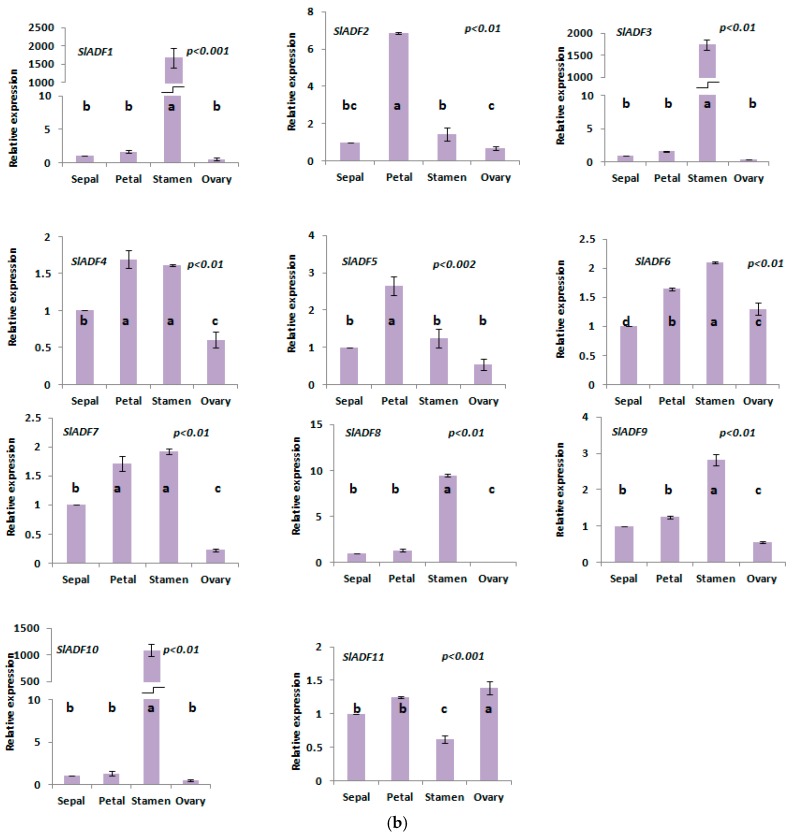
Tissue specificity of tomato *ADF* gene expression: (**a**) Expression levels of the 11 *ADF* genes via quantitative PCR in different organs; root, stem, leaf, whole flower and fruit at six developmental stages (1 cm: 1 centimeter sized fruit; IM: immature fruit; MG: mature green fruit; B: breaker; B + 5: five days after breaker; B + 10: 10 days after breaker). The standard error of the means of three independent replicates is represented by the error bars. *p* values indicate statistically significant variations of expression. Different lowercase letters (a, b, c, etc.) indicate statistically significant difference. (**b**) Expression levels of the 11 *ADF* genes via quantitative PCR in the different floral organs; sepal, petal, stamen and ovary. The standard error of the means of three independent replicates is represented by the error bars. *p* values indicate the statistically significant variations of expression. Mean values at different sampling points are represented by different letters.

The expression patterns of the different tomato *ADF* genes are shown in Figure 4a. Among the 11 genes, *SlADF2*, *SlADF4*, *SlADF5*, *SlADF7*, *SlADF8*, *SlADF9* and *SlADF11* were expressed at variable levels in all organs examined including the different stages of fruits. *SlADF2* and *SlADF11* were highly expressed in flowers compared to other organs. The expression of *SlADF2* was about three times higher and that of *SlADF11* was about four times higher in flowers than fruits at ten days after the breaker stage. *SlADF1* and *SlADF10* showed flower-specific expression whereas *SlADF6* was preferentially expressed in roots. *SlADF3* was predominantly expressed in flowers and in fruits at the immature and mature green stage. When the expression profiles at the different fruit developmental stages was compared, we found that *SlADF3*, *SlADF9* and *SlADF11* showed significantly higher expression at the early stages, mainly in the immature and mature green stage, compared to 5 and 10 days after breaker stage. By contrast, *SlADF5* and *SlADF8* strongly expressed at 5 days after the breaker stage, the peak of fruit ripening, compared to other stages of fruit development. Expression of the *ADF* genes was further investigated in different parts of the flower, sepals, petals, stamen and ovary (Figure 4b). *SlADF1*, *SlADF3* and *SlADF10* were predominately expressed in stamen compared to other flower parts. *SlADF6*, *SlADF7*, *SlADF8* and *SlADF9* also had higher expression levels in the stamen compared to other parts of the flower. *SlADF2* had about six times higher expression in the petals than in the sepals, stamen and ovary. Expression of *SlADF4* and *SlADF5* was higher in the petals and stamen compared to the sepals and ovary.

### 3.5. Expression Analysis of Tomato ADF Genes under Different Abiotic Stresses

**Figure 5:**
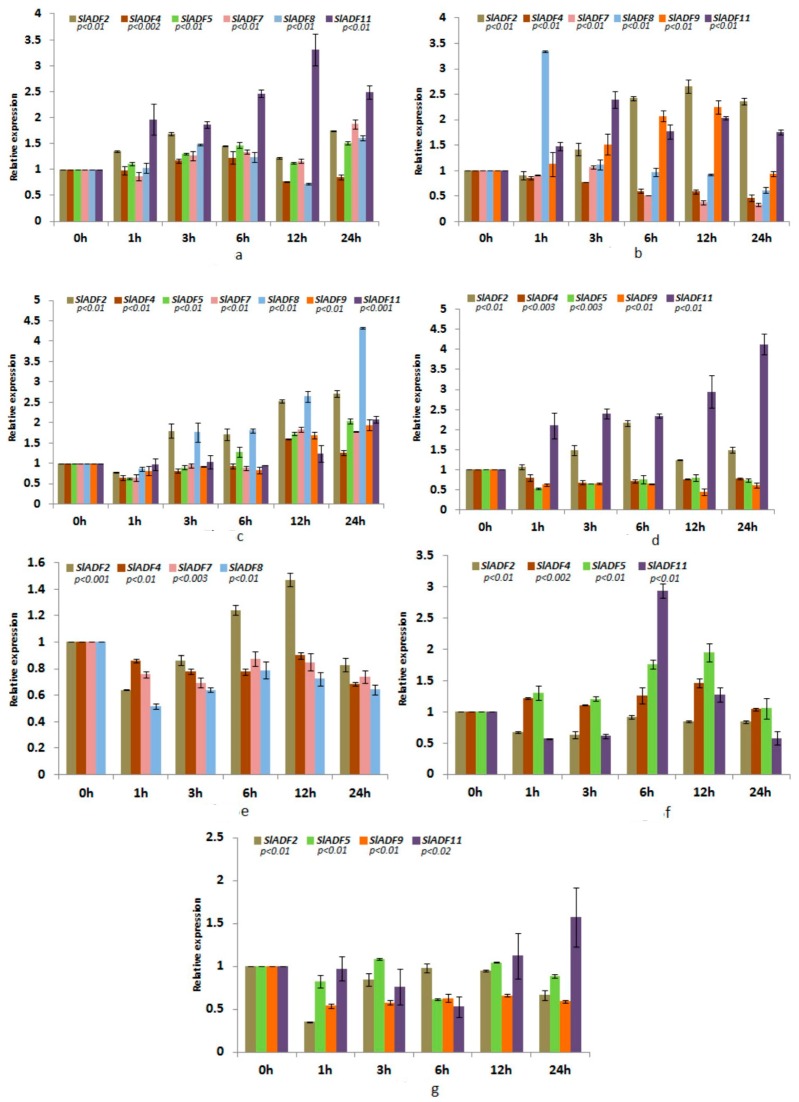
Stress-related *ADF* gene expression in tomato. Expression levels of the 11 *ADF* genes via quantitative PCR under different stresses: (**a**) cold; (**b**) heat; (**c**) drought; (**d**) NaCl; (**e**) abscisic acid (ABA); (**f**) Jasmonic acid (JA); and (**g**) wounding. The standard error of the means of three independent replicates is represented by the error bars. P-values indicate the statistically significant variations of expression. Mean values at different sampling points are separated by different letters.

Seven of the tomato *ADF* genes (*SlADF2*, *SlADF4*, *SlADF5*, *SlADF7*, *SlADF8*, *SlADF9* and *SlADF11*) were differentially regulated by the abiotic stresses examined in this study (Figure 5a–g).

#### 3.5.1. Cold Stress

The expression patterns of the cold-treated samples are shown in Figure 5a. The expression of *SlADF2*, *SlADF5* and *SlADF11* was significantly up-regulated under cold stress compared to the control. *SlADF4* showed a slight up-regulation until 6 h of cold treatment but was then down-regulated in the remaining period compared to the control. The expression of *SlADF7* remained increased after 1 h and until 24 h of treatment compared to the control. The expression of *SlADF8* increased until 6 h of treatment, then decreased at 12 h followed by another increase at 24 h compared to the control.

#### 3.5.2. Heat Stress

The expression patterns of the heat-treated samples are shown in Figure 5b. The expression of *SlADF2* and *SlADF11* was significantly up-regulated in response to heat treatment compared to the control while *SlADF4* was down-regulated. The expression of *SlADF7* was reduced compared to the control with the exception of a slight increase at 3 h. Markedly higher expression of *SlADF8*, around three-fold higher than the control, was found after 1 h of treatment while the expression level remained similar to the control for the remaining treatment period. *SlADF9* was gradually up-regulated during treatment until 12 h followed by a decrease at 24 h of treatment.

#### 3.5.3. Drought Stress

The expression patterns of the drought-stressed samples are shown in Figure 5c. The expression of *SlADF2* was gradually up-regulated during drought stress treatment. *SlADF4*, *SlADF7*, *SlADF9* and *SlADF11* were slightly down-regulated until 6 h followed by an increase at 12 h and 24 h of treatment compared to the control. The expression of *SlADF5* was down-regulated until 3 h and then gradually increased during the remaining treatment period compared to the control. The expression level of *SlADF8* significantly increased after 1 h and around four times higher expression was observed after 24 h of treatment compared to the control.

#### 3.5.4. NaCl Stress

The expression patterns of the NaCl-treated samples are shown in Figure 5d. Under NaCl stress, the expression levels of *SlADF2* and *SlADF11* were significantly up-regulated while *SlADF4*, *SlADF5* and *SlADF9* were down-regulated compared to the control.

#### 3.5.5. ABA Treatment

The expression patterns of the ABA-treated samples are shown in Figure 5e. The expression levels of *SlADF4*, *SlADF7* and SlADF*8* were down-regulated under ABA treatment compared to the control. The expression of *SlADF2* increased only at 6 h and 12 h of treatment compared to the control.

#### 3.5.6. JA Treatment

The expression patterns of the JA-treated samples are shown in Figure 5f. The expression levels of *SlADF4* and *SlADF5* were slightly up-regulated while *SlADF2* was down-regulated under JA treatment compared to the control. *SlADF11* was highly expressed, around three-fold higher compared to the control, after 6 h of treatment while its expression was reduced during the remainder of the treatment period.

#### 3.5.7. Wounding

The expression patterns of the wounded samples are shown in Figure 5g. *SlADF2* and *SlADF9* were slightly down-regulated during wounding stress compared to the control. *SlADF5* showed slight up-regulation compared to the control at 3 h and 12 h of treatment but was down-regulated during the other time periods. *SlADF11* was gradually down-regulated until 6 h followed by an increase at 12 h and 24 h of treatment compared to the control.

## 4. Discussion

The ADF protein family is highly conserved both structurally and functionally, even in distantly related species like yeast and mammals and believed to have a similar function in dynamic actin cytoskeleton organization and regulation [26]. The N-terminus serine residue has been found to regulate the activity of ADF proteins both in vertebrates and in maize [27,28,29]. A calcium-dependent calmodulin-like domain protein kinase is involved in the phosphorylation of the serine residue [10]. The phosphorylation of ADF may be linked to the calcium signaling pathway and thereby reorganize the cytoskeleton in response to environmental and developmental signals. Except SlADF2, all other 10 SlADF proteins had a conserved serine residue followed by a glycine residue in their N-terminus region, which might be phosphorylated as in other known plant ADF proteins (Figure 2) [30,31]. The N-terminus serine of SlADF2 was substituted by a threonine residue, which could probably be phosphorylated by a regulatory kinase (Figure 2). The structural similarity of the tomato *ADF* genes and proteins to those of Arabidopsis and rice suggested that the SlADFs have similar actin binding and severing activity in dynamic actin cytoskeleton reorganization. The phylogenetic classification of 11 tomato ADF proteins clearly confirmed the existence of the four previously identified groups and was consistent with the findings of Feng et al. (2006) [8]. The high homology of the tomato ADFs with those of Arabidopsis and rice in the phylogenetic tree suggests that they evolved from common ancestors and also indicates their functional similarities in flowering plants. Due to considerable variation in the sequences, some of the branch bootstrap values in the phylogenetic tree were found to be insignificant.

Actin binding proteins may changes the actin cytoskeleton rearrangement through different intracellular signaling processes. The ADF/cofilin proteins bind to F-actin by severing the actin filaments and also by dissociating the globular actin (G-actin) from the pointed or minus end of actin and thereby enhancing the filament turnover rate [12,32,33,34,35]. Filamentous actin (F-actin) has been reported to be involved in pollen tube growth, trichome morphogenesis, root hair tip growth and fiber elongation [36,37]. The F-actin cytoskeleton is also involved in flower induction during petal development by transporting signals between cells [38]. In *Gossypium hirsutum*, the down-regulation of *GhADF1* accelerates cotton fiber length and strength as a result of formation of more abundant F-actin filaments [39]. The differential expression of *SlADF* genes in different tissues implies that functional variation might have arisen during evolution. The expression of different *SlADF* genes was also consistent with the ancient classification of *ADF* family genes [7]. The predominant expression of *SlADF1*, *SlADF3* and *SlADF10* (Figure 4a,b) in flowers, especially in the stamen suggests that these three genes might have an association with flower and pollen development and other regulatory factors. Members of plant *ADFs* have been reported to be involved in the flower induction signaling pathway and in pollen tube growth through the rearrangement of the actin cytoskeleton [16,31,36]. AtADF proteins regulate F-actin organization and have important functions in physiological processes including cell and organ expansion in Arabidopsis [13].

Plants absorb water and minerals through their roots for growth and development; accordingly, proper development of roots and root hairs is crucial for adaptation and vigor. Active accumulation of ADF was found in the root tip region to sustain the turnover of the F-actin that is required for the cell elongation [37] and it has been reported that *ADFs* are associated with root formation and root hair development in plants [14,37]. *SlADF6* was predominately expressed in roots (Figure 4a), suggesting that as a regulator of actin dynamics, *SlADF6* could reset the cytoskeletal network during root development of tomato and thereby might play a role in root formation and development. The other seven tomato *ADF* genes were expressed at various levels in different organs, suggesting that they might have various regulatory functions in tomato growth and development. The function of *ADF* genes in plant growth and development has been confirmed in other species. For instance, reduced expression of AtADF1 stimulated cell expansion and organ growth by promoting actin cable formation, whereas overexpression of AtADF1 resulted in the disappearance of thick actin cables and reduced cell and organ growth [13]. In addition, shorter hypocotyls and decreased root hair formation was observed upon ectopic expression of *Gossypium barbadense ADF1* in tobacco [40]. However, it remains to be explored whether *SlADF* genes regulate growth and development of tomato by organizing actin cytoskeleton structure.

Actin binding proteins (ABPs) control the functional link between the F-actin and the auxin flow [41]. In addition to cell elongation, auxins regulate fruit set, growth and ripening, apical dominance, and leaf senescence, as well as playing an essential role in tropic responses to light and gravity [42]. In tobacco, ADF2 might control the dynamics of cortical actin filaments, which is required for functional auxin-dependent signaling with respect to synchronized cell division [43,44]. Tomato fruit attains its final size through successive cell divisions until 1 cm fruit stage, following by cell expansion until the immature and mature green fruit stage. The cell expansion phases, IM and MG, are the longest phases of fruit development. Depending on the cultivar, about 90% of fruit weight increase occurs during these phases [45]. During the cell expansion phase, auxin produced from the seeds and surrounding tissues accelerates fruit growth [46]. The significantly higher expression of *SlADF11* in the early developmental, 1 cm, immature (IM) and mature green (MG) stages, and of *SlADF3*, *SlADF9* and *SlADF11* in the IM and MG stages compared to the ripening stages suggested that those *SlADF* genes might have active roles controlling actin filaments during fruit set and expansion through the auxin signaling pathway. Ripening of tomato fruits is related to the level of ethylene production [47]. Ethylene production starts to increase at the breaker stage and reaches its maximum level at the full ripening stage [47]. *SlADF5* and *SlADF8* were predominately expressed at five days after breaker stage, the peak of ripening, indicating their relatedness in fruit ripening through the ethylene biosynthesis pathway. Moreover, auxin- and ethylene-responsive cis-elements were also found in most of the *SlADF* genes, indicating their possible function in the early development and ripening of tomato fruit. Cell expansion and organ growth related functions of *ADF* genes are also found in Arabidopsis [31]. In maize, *ZmADF3* is expressed to different levels in various tissues and developmental stages during kernel development, in terms of both RNA and proteins levels, implicating them as having important roles in kernel development [48]. These possible functions of *SlADFs* in fruit enlargement and ripening should be the foundation for further functional analysis of this gene family in tomato.

The actin cytoskeleton is reorganized by ADF in response to external and internal stimuli, and thereby changes the morphology of the cell. Accordingly, cytoskeleton rearrangement is used as a marker for various stress signaling pathways such as salt, cold, gravity, osmotic pressure, wounding and pathogen attack [49,50,51,52]. Together, ADF, actin-related protein 2 (ARP2) and actin-binding protein 29 (ABP29) are involved in actin cytoskeleton remodeling under salt stress conditions [53]. The rapid reorganization of the cytoskeleton structure promotes salinity tolerance in cotton roots [54]. The differential assembly of actin filaments under salt stress is crucial for salt stress tolerance in Arabidopsis [54]. The possible association of ADF in dynamic cytoskeleton rearrangement may provide a clue to abiotic stress tolerance mechanisms in plants. Proteomics studies suggested the involvement of *OsADF1* and *OsADF3* in drought tolerance in an upland rice variety [55,56]. Marked variation in the expression levels of different tomato *ADF* genes was observed under the different stresses. Based on this differential expression we propose that the SlADF proteins might remodel the cytoskeleton structure in response to different stress signals in cell and this remodeling might be important for the enhancement of stress tolerance in tomato. Notably, abiotic stress-responsive cis-regulatory elements were found in most of the *SlADF* gene sequences (Supplementary Table S5) supporting the likelihood of abiotic stress tolerance-related functions for the tomato *ADF* genes. In transgenic Arabidopsis, the ectopic over-expression of OsADF3 conferred mannitol- and drought-stress tolerance by promoting the germination rate, primary root length and survival of seedlings [57]. Further screening and functional analysis of *SlADF* genes are needed to explore the possible relationship between actin remodeling and the physiological functions of *SlADF* genes triggered by different stresses.

## 5. Conclusions

ADFs regulate actin cytoskeletal organization in response to different cellular activities and cell signaling events. In addition to cytoskeleton reorganization, ADFs are involved in organ development in plants. This study represents the first comprehensive study of this important gene family in a vegetable crop. The 11 identified *ADF* genes were phylogenetically classified into four groups showing consistency with their organ-specific expression patterns. Three genes that were preferentially expressed in the flower and especially the stamen, *SlADF1*, *SlADF3* and *SlADF10*, are likely associated with flower and pollen development. The unique expression of *SlADF6* in roots makes it a good candidate gene for functioning in root development of tomato. We also analyzed the expression of tomato *ADFs* under different abiotic and phytohormone stresses. The expressions of *SlADF2*, *SlADF4*, *SlADF5*, *SlADF6*, *SlADF7*, *SlADF8*, *SlADF9* and *SlADF11* were induced by the different stresses, suggesting that these genes may have stress tolerance functions in tomato. These results will be helpful for further functional validation of candidate genes in relation to vegetative growth, reproductive development and stress tolerance of tomato.

## Figures and Tables

**Table 1:** In silico analysis of the *actin depolymerizing factor* (*ADF*) genes collected from the *Solanum lycopersicum* database.

Gene Name	Locus Name	ORF (bp)	Chromosomal Location	Protein					Subcellular Localization	No. of Introns
				Length (aa)	ADF Domain Start–End (aa)	MW (KDa)	pI	GRAVY		
*SlADF1*	Solyc01g094400	414	1	137	12–137	15.63	6.13	−0.437	Extracellular	1
*SlADF2*	Solyc01g111380	432	1	143	16–143	16.44	8.69	−0.397	Extracellular	2
*SlADF3*	Solyc03g025750	414	3	137	12–137	15.80	5.12	−0.442	Extracellular	1
*SlADF4*	Solyc04g011370	432	4	143	12–139	16.49	5.33	−0.469	Extracellular	1
*SlADF5*	Solyc06g005360	414	6	137	12–137	15.84	6.15	−0.350	Extracellular	1
*SlADF6*	Solyc06g035980	414	6	137	12–137	15.79	5.50	−0.293	Extracellular	1
*SlADF7*	Solyc09g010440	420	9	139	12–139	16.00	6.73	−0.465	Extracellular	1
*SlADF8*	Solyc09g072590	450	9	145	18–145	16.69	7.77	−0.594	Extracellular	2
*SlADF9*	Solyc09g090110	456	9	145	18–145	16.84	6.74	−0.598	Extracellular	2
*SlADF10*	Solyc10g017550	420	10	139	12–139	15.98	5.63	−0.524	Extracellular	1
*SlADF11*	Solyc10g084660	420	10	139	12–139	16.11	5.29	−0.523	Extracellular	1

Abbreviations: ORF, open reading frame; bp, base pair; aa, amino acid; MW, molecular weight; KDa, kilo Dalton; pI, iso-electric point; GRAVY, grand average of hydropathy.

## Supplementary Materials

The following are available online at www.mdpi.com/2073-4425/7/10/79/s1. Figure S1: Schematic representation of the conserved motif identified in the tomato ADF proteins. Different colors represent different motifs. The motif order in the figure corresponds to their position in the individual protein sequences. The name of each member is shown on the left side of the figure, Figure S2: Positions of the 11 *ADF* genes along the 12 tomato chromosomes. The black dotted lines represent the duplicated genes in the genome. Chromosome sizes and gene positions were estimated using the scale in megabases (Mb) to the left of the figure, Table S1: Primer sequences used for qPCR analysis of tomato *ADF* genes, Table S2: Similarity analysis of the tomato *ADF* gene family, Table S3: Sequence identity among the 11 ADF proteins of tomato, Table S4: Segmentally duplicated genes among the 11 tomato *ADF* genes, Table S5: Putative *cis*-elements of more than 5 bp identified in the 11 tomato *ADF* genes using the PlantCARE database, Additional File 1: Putative protein sequences of tomato, Arabidopsis, rice and cotton used in in silico analysis.
