# Supplementary material for: Genome-Wide Identification, Characterization and Expression Profiling of ADF Family Genes in Solanum lycopersicum L
Source: Genes (Basel). 2016 Sep 29;7(10):79. doi: 10.3390/genes7100079 (PMC5083918; doi:10.3390/genes7100079)
Supplement: Supplementary file 1 [file genes-07-00079-s001.zip › genes-142380-supplementary-layout/genes-142380-Supplementary materials-layout.docx]

Supplementary Materials: Genome-Wide Identification, Characterization and Expression Profiling of *ADF* Family Genes in *Solanum lycopersicum* L.

Khadiza Khatun, Arif Hasan Khan Robin, Jong-In Park, Chang Kil Kim, Ki-Byung Lim, Min-Bae Kim, Do-Jin Lee, Ill Sup Nou and Mi-Young Chung


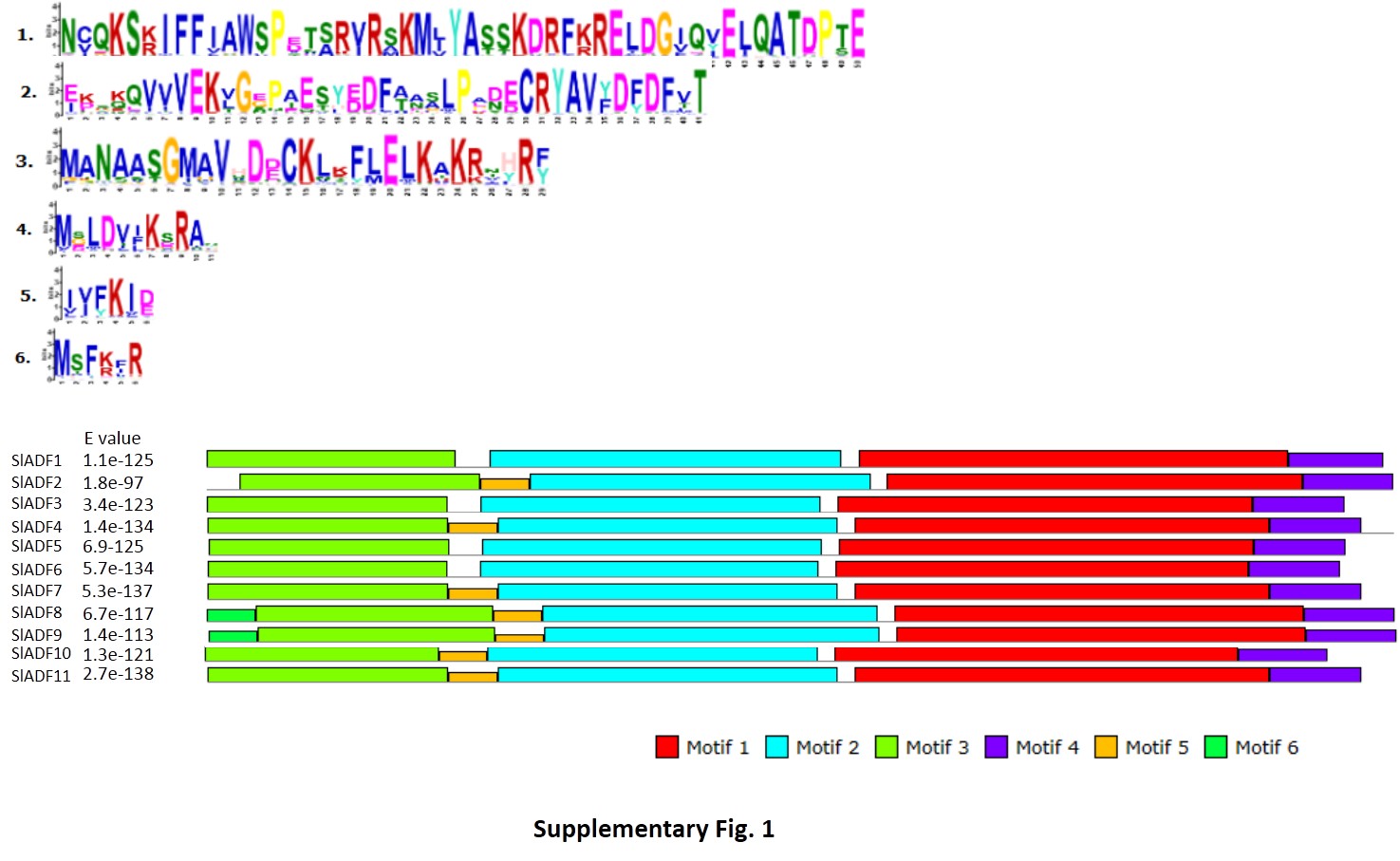


**Figure S1.** Schematic representation of the conserved motif identified in the tomato actin depolymerizing factor (ADF) proteins. Different colors represent different motifs. The motif order in the figure corresponds to their position in the individual protein sequences. The name of each member is shown on the left side of the figure.


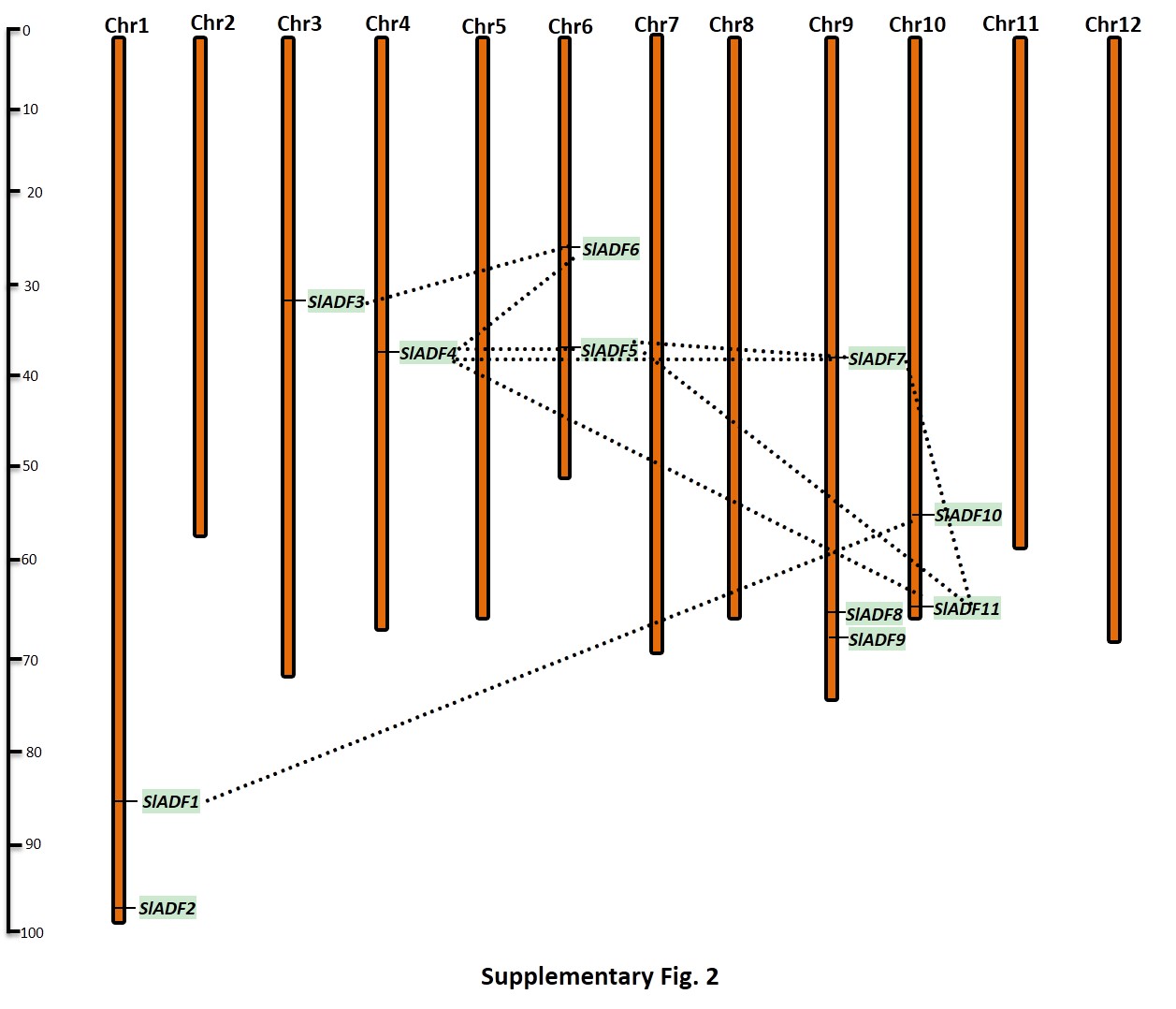


**Figure S2.** Positions of the 11 *actin depolymerizing factor* (*ADF*) genes along the 12 tomato chromosomes. The black dotted lines represent the duplicated genes in the genome. Chromosome sizes and gene positions were estimated using the scale in megabases (Mb) to the left of the figure.

**Table S1**. Primer sequences used for q-PCR analysis of tomato *ADF* genes.

| **Gene ID** | **Primer Sequence** | | **Product length(bp)** |
| --- | --- | --- | --- |
|  | **Forward** | **Reverse** |  |
| *SlADF1* | TTCGATGGTGTTCAGGTTGA | CCATCAAGAAAAAGGGCCTA | 160 |
| *SlADF2* | TCCAACTGAGATGGGAATGG | TTTGCCCTCTAAGTGGAGTCA | 150 |
| *SlADF3* | TGGTCACCTGAATCATCAACA | GACTCAGTAGGCTCGCGATT | 156 |
| *SlADF4* | ACAGAGATGGGGCTTGATGT | AAGGTGATTGGGAACGACAA | 150 |
| *SlADF5* | GCCGTGCCAACTAAATAAACA | TGAGTTCCACACACAAACATCA | 189 |
| *SlADF6* | GAATTGCAAGCAACAGATCCT | TCCAGAAATGATCAGGGTGA | 195 |
| *SlADF7* | ATTAAGAGCCGCGCCAACTA | TGTAGAGCACTTGGCAATCAA | 151 |
| *SlADF8* | AAGTGCTGAAAGACCGTGCT | AGAAAATTGGCAACCACTGA | 172 |
| *SlADF9* | CAGAGGGACAAATGCATCCT | TCAGGAAGAGCTGCAGTGAA | 189 |
| *SlADF10* | TGAGTTTGGATACCTTTGTTGG | TATTTCTGGGCCAGCGACTAA | 169 |
| *SlADF11* | TTCCTGCTGATGAATGCAGA | AGTTGGATCAGTAGCCTGCAA | 203 |

**Table S2.** Similarity analysis of *ADF* gene family of *Solanum lycopersicum****^a^***.

| **Sl. No.** | **Name of gene** | **Top matched ADF clone** | **Name of matched protein** | **Identity (%)** | **E value** | **Matched species** | **References** |
| --- | --- | --- | --- | --- | --- | --- | --- |
| 1 | *SlADF1* | BAB10533 | Actin depolymerizing factor-like | 75 | 1e-69 | *Arabidopsis thaliana* | [1] |
|  |  | XP_007051847 | Actin depolymerizing factor 11 | 77 | 1e-73 | *Theobroma cacao* | [2] |
| 2 | *SlADF2* | XP_007041872 | Actin depolymerizing factor 5 | 88 | 2e-88 | *Theobroma cacao* | [2] |
|  |  | NP_565390 | Actin depolymerizing factor 5 | 82 | 1e-80 | *Arabidopsis thaliana* | [3] |
| 3 | *SlADF3* | AAL91666 | Pollen specific actin-depolymerizing factor 1 | 88 | 3e-84 | *Nicotiana tabacum* | [4] |
|  |  | AAM61402 | Actin depolymerizing factor-like | 88 | 2e-83 | *Arabidopsis thaliana* | [5] |
| 4 | *SlADF4* | XP_002314195 | Actin-depolymerizing factor family protein | 90 | 1e-87 | *Populus trichocarpa* | [6] |
|  |  | ADV04049 | Actin depolymerizing factor 4 | 88 | 1e-87 | *Hevea brasiliensis* | [7] |
| 5 | *SlADF5* | XP_002314194 | Actin-depolymerizing factor 3 family protein | 85 | 1e-81 | *Populus trichocarpa* | [6] |
|  |  | XP_007016496 | Actin depolymerizing factor 4 | 83 | 1e-79 | *Theobroma cacao* | [2] |
| 6 | *SlADF6* | XP_007038697 | Actin depolymerizing factor 7 | 83 | 1e-80 | *Theobroma cacao* | [2] |
|  |  | NP_194289 | Actin depolymerizing factor 7 | 85 | 4e-83 | *Arabidopsis thaliana* | [8] |
| 7 | *SlADF7* | XP_002314195 | Actin-depolymerizing factor family protein | 88 | 2e-86 | *Populus trichocarpa* | [6] |
|  |  | ABD66504 | Actin depolymerizing factor 8 | 88 | 1e-85 | *Gossypium hirsutum* | [9] |
| 8 | *SlADF8* | NP_001294893 | Actin-depolymerizing factor | 82 | 1e-75 | *Jatropha curcas* | [10] |
|  |  | XP_007035112 | Actin depolymerizing factor 6 | 79 | 1e-80 | *Theobroma cacao* | [2] |
| 9 | *SlADF9* | XP_002300779 | Actin-depolymerizing factor 6 family protein | 83 | 1e-75 | *Populus trichocarpa* | [6] |
|  |  | XP_007035112 | Actin depolymerizing factor 6 | 86 | 2e-86 | *Theobroma cacao* | [2] |
| 10 | *SlADF10* | XP_002303579 | Actin-depolymerizing factor family protein | 76 | 1e-69 | *Populus trichocarpa* | [6] |
|  |  | XP_007040194 | Actin depolymerizing factor 8 | 74 | 1e-72 | *Theobroma cacao* | [2] |
| 11 | *SlADF11* | XP_007016496 | Actin depolymerizing factor 4 | 91 | 2e-89 | *Theobroma cacao* | [2] |
|  |  | ABX79380 | Actin-depolymerizing factor | 89 | 2e-87 | *Gossypium barbadense* | [11] |

^a^Analyzed using BLAST from NCBI, http://www.ncbi.nlm.nih.gov/BLAST/.

**Table S3.** Sequence identity among the 11 ADF proteins of tomato.

|  | A | B | C | D | E | F | G | H | I | J | K |
| --- | --- | --- | --- | --- | --- | --- | --- | --- | --- | --- | --- |
| SlADF1(A) | 100 |  |  |  |  |  |  |  |  |  |  |
| SlADF2(B) | 54 | 100 |  |  |  |  |  |  |  |  |  |
| SlADF3(C) | 74 | 50 | 100 |  |  |  |  |  |  |  |  |
| SlADF4(D) | 66 | 54 | 70 | 100 |  |  |  |  |  |  |  |
| SlADF5(E) | 64 | 51 | 69 | **81** | 100 |  |  |  |  |  |  |
| SlADF6(F) | 72 | 54 | **84** | 78 | 77 | 100 |  |  |  |  |  |
| SlADF7(G) | 66 | 58 | 68 | **84** | 79 | 76 | 100 |  |  |  |  |
| SlADF8(H) | 56 | 57 | 58 | 60 | 56 | 59 | 64 | 100 |  |  |  |
| SlADF9(I) | 55 | 58 | 55 | 55 | 54 | 57 | 61 | 79 | 100 |  |  |
| SlADF10(J) | **85** | 51 | 67 | 67 | 61 | 65 | 66 | 57 | 53 | 100 |  |
| SlADF11(K) | 67 | 57 | 69 | **87** | 82 | 77 | **89** | 63 | 61 | 69 | 100 |

**Table S4.** Segmentally duplicated genes among the 11 tomato *ADF* genes.

| **Gene name** | **Duplicated genes** | **Query cover (%)** | **Identity (%)** | ***E*-value** |
| --- | --- | --- | --- | --- |
| *SlADF1* | *SlADF10* | 98 | 88 | 5e-93 |
| *SlADF3* | *SlADF6* | 99 | 85 | 9e-90 |
| *SlADF4* | *SlADF11* | 97 | 88 | 8e-96 |
|  | *SlADF7* | 97 | 84 | 3e-92 |
|  | *SlADF5* | 97 | 83 | 1e-87 |
|  | *SlADF6* | 96 | 80 | 9e-86 |
| *SlADF5* | *SlADF4* | 100 | 83 | 1e-87 |
|  | *SlADF11* | 100 | 83 | 5e-87 |
|  | *SlADF7* | 100 | 81 | 5e-85 |
| *SlADF6* | *SlADF3* | 99 | 85 | 9e-90 |
|  | *SlADF4* | 99 | 80 | 8e-86 |
| *SlADF7* | *SlADF11* | 100 | 89 | 8e-96 |
|  | *SlADF4* | 100 | 84 | 3e-92 |
|  | *SlADF5* | 100 | 81 | 5e-85 |
| *SlADF11* | *SlADF4* | 100 | 88 | 8e-96 |
|  | *SlADF7* | 100 | 89 | 8e-96 |
|  | *SlADF5* | 100 | 83 | 5e-87 |

**Reference**

1. Sato, S.; Nakamura, Y.; Kaneko, T.; Katoh, T.; Asamizu, E.; Kotani, H.; Tabata, S. Structural analysis of arabidopsis thaliana chromosome 5. X. sequence features of the regions of 3,076,755 bp covered by sixty P1 and TAC clones. *DNA Res*. **2000**, *7*, 31–63.
2. Motamayor, J.C.; Mockaitis, K.; Schmutz, J.; Haiminen, N.; Livingstone, III.D.; Cornejo, O.; Saski, C. The genome sequence of the most widely cultivated cacao type and its use to identify candidate genes regulating pod color. *Genome biol.* **2013**, doi:10.1186/gb-2013-14-6-r53
3. Lin, X.; Kaul, S.; Rounsley, S.; Shea, T.P.; Benito, M.I.; et al. Sequence and analysis of chromosome 2 of the plant *Arabidopsis thaliana*. *Nature* **1999**, *16,* 761–768.
4. Chen, C.Y.; Wong, E.I.; Vidali, L.; Estavillo, A.; Hepler, P.K.;Wu, H.M.; Cheung, A.Y. The regulation of actin organization by actin-depolymerizing factor in elongating pollen tubes. *Plant Cell* **2002**, *14*, 2175–2190.
5. Haas, B.J.; Volfovsky, N.; Town, C.D.; Troukhan, M.; Alexandrov, N.; Feldmann, K.A.; Flavell, R.B.; White, O.; Salzberg, S.L. Full-length messenger RNA sequences greatly improve genome. *Genome Biol*. **2002**, *3*, 1.
6. Tuskan, G.A.; Difazio, S.; Jansson, S.; Bohlmann, J.; Grigoriev, I.; Hellsten, U.; Putnam, N.; Ralph, S.; Rombauts, S.; Salamov, A.; et al. The genome of black cottonwood, *Populus trichocarpa* (torr. & gray). *Science* **2006**, *313*, 1596–1604.
7. Li, H.; Qin, Y.; Xiao, X.; Tang, C. Screening of valid reference genes for real-time RT-PCR data normalization in *Hevea brasiliensis* and expression validation of a sucrose transporter gene HbSUT3. *Plant Sci*. **2011**, *181*, 132-9.
8. Mayer, K.; Schüller, C.; Wambutt, R.; Murphy, G.; Volckaert, G.; Pohl, T.; Düsterhöft, A.; Stiekema, W.; Entian, K.; Terryn, N. Sequence and analysis of chromosome 4 of the plant *Arabidopsis thaliana*. *Nature* **1999**, *402*, 769–77.
9. Li, X.B.; Xu, D.; Wang, X.L.; Huang, G.Q.; Luo, J.; Li, D.D.; Zhang, Z.T.; Xu, W.L. Three cotton genes preferentially expressed in flower tissues encode actin-depolymerizing factors which are involved in F-actin dynamics in cells. *J. Exp. Bot.* **2010**, *61*, 41–53.
10. Zhang, L.; Zhang, C.; Wu, P.; Chen, Y.; Li, M.; Jiang, H.; Wu, G. Global analysis of gene expression profiles in physic nut (*Jatropha curcas L.)* seedlings exposed to salt stress. *PloS ONE* **2014**, *9*, e97878.
11. Chi, J.; Wang, X.; Zhou, H.; Zhang, G.; Sun, Y.; Li, Z.; Ma, Z. Molecular cloning and characterization of the actin-depolymerizing factor gene in *Gossypium barbadense*. *Genes Genet. Syst*. **2008**, *83*, 383–91.
